# Protected Geographical Indication Discrimination of Zhejiang and Non-Zhejiang *Ophiopogonis japonicus* by Near-Infrared (NIR) Spectroscopy Combined with Chemometrics: The Influence of Different Stoichiometric and Spectrogram Pretreatment Methods

**DOI:** 10.3390/molecules28062803

**Published:** 2023-03-20

**Authors:** Qingge Ji, Chaofeng Li, Xianshu Fu, Jinyan Liao, Xuezhen Hong, Xiaoping Yu, Zihong Ye, Mingzhou Zhang, Yulou Qiu

**Affiliations:** 1 Zhejiang Provincial Key Laboratory of Biometrology and Inspection & Quarantine, College of Life Science, China Jiliang University, Hangzhou 310018, China; qinggeji1997@163.com (Q.J.); lcf609162059@163.com (C.L.); yxp@cjlu.edu.cn (X.Y.); zhye@cjlu.edu.cn (Z.Y.); zmzcjlu@cjlu.edu.cn (M.Z.); yulou@cjlu.edu.cn (Y.Q.); 2 Business and Trade Branch, Zhejiang Yuying College of Vocational Technology, Hangzhou 310018, China; 3 College of Quality & Safety Engineering, China Jiliang University, Hangzhou 310018, China; xzhong@cjlu.edu.cn

**Keywords:** *Ophiopogon japonicus*, protected geographical indication discrimination, chemometrics, spectral pretreatment

## Abstract

This paper presents a method for the protected geographical indication discrimination of Ophiopogon japonicus from Zhejiang and elsewhere using near-infrared (NIR) spectroscopy combined with chemometrics. A total of 3657 *Ophiopogon japonicus* samples from five major production areas in China were analyzed by NIR spectroscopy, and divided into 2127 from Zhejiang and 1530 from other areas (‘non-Zhejiang’). Principal component analysis (PCA) was selected to screen outliers and eliminate them. Monte Carlo cross validation (MCCV) was introduced to divide the training set and test set according to a ratio of 3:7. The raw spectra were preprocessed by nine single and partial combination methods such as the standard normal variable (SNV) and derivative, and then modeled by partial least squares regression (PLSR), a support vector machine (SVM), and soft independent modeling of class analogies (SIMCA). The effects of different pretreatment and chemometrics methods on the model are discussed. The results showed that the three pattern recognition methods were effective in geographical origin tracing, and selecting the appropriate preprocessing method could improve the traceability accuracy. The accuracy of PLSR after the standard normal variable was better, with R^2^ reaching 0.9979, while that of the second derivative was the lowest with an R^2^ of 0.9656. After the SNV pretreatment, the accuracy of the training set and test set of SVM reached the highest values, which were 99.73% and 98.40%, respectively. The accuracy of SIMCA pretreated with SNV and MSC was the highest for the origin traceability of *Ophiopogon japonicus*, which could reach 100%. The distance between the two classification models of SIMCA-SNV and SIMCA-MSC is greater than 3, indicating that the SIMCA model has good performance.

## 1. Introduction

*Ophiopogon japonicus* is the dried tuberous root of *Ophiopogon japonicus* (Thunb.) Ker.-Gawl. (family Liliaceae), mainly produced in Sichuan, Zhejiang, Hubei and Anhui provinces [1]. Its main active components include steroid saponins, high isoflavones, polysaccharides, amino acids, volatile oils, trace elements, etc. [2]. Modern pharmacological and clinical studies have shown that *Ophiopogon japonicus* has, for example, antitumor, anti-inflammatory, hypoglycemic, and antiaging effects, and it can be used in the treatment of diabetes [3,4]. *Ophiopogon japonicus*, compatible with red ginseng and schisandra chinensis, can be made into Shengmai Yin, which is widely used in the treatment of acute myocardial infarction, cardiogenic shock, arrhythmia, and other heart diseases [5]. *Ophiopogon japonicus* is not only a kind of traditional Chinese medicine (TCM), but is also a typical homologous product of medicine and food. It has been used as a health food for a long time to enhance human immunity and soothe the mind [6].

At present, *Ophiopogon japonicus* is mostly cultivated in China, with common cultivation areas in Sichuan, Zhejiang, Hubei, Anhui and other provinces. The *Ophiopogonis japonicus* of Zhejiang is one of the eight traditional medicinal materials in Zhejiang. While the *Ophiopogonis japonicus* of Zhejiang is three years old, all the other *Ophiopogonis japonicus* are annual, which makes the quality of Zhejiang *Ophiopogonis japonicus* much higher than that produced elsewhere [7]. Lyu et al., analyzed the chemical composition of *Ophiopogon japonicus* with UPLC/Q-TOF MS, and found that there were significant differences in the chemical composition of *Ophiopogon japonicus* from different production origins [8]. The content of isoflavone compounds in the *Ophiopogon japonicus* of Zhejiang is higher. It also contains borneoside, which has the function of anti-myocardial ischemia [9] and is not found in other species [10]. To sum up, we know that the quality of the *Ophiopogon japonicus* of Zhejiang is better than that of other producing areas, which makes its price much higher [11]. According to survey statistics, the price of *Ophiopogon japonicus* of Zhejiang is about 500–600 yuan per kg, 5 times higher than that of non-Zhejiang *Ophiopogon japonicus*. As such, non-Zhejiang *Ophiopogon japonicus* often appears in the market as from Zhejiang. Most of the *Ophiopogon japonicus* on the market is sold in powder form, which makes it very difficult to distinguish and discriminate its origins. The discrimination of the protected geographical indication of *Ophiopogon japonicus* can not only combat fake and shoddy products, but can also provide reference for the traceability of other traditional Chinese medicines.

There are many common methods for the origin tracing of traditional Chinese medicines, including stable isotope techniques [12,13], high-performance liquid chromatography (HPLC) analysis [14], DNA barcoding techniques [15], near-infrared (NIR) spectroscopy [16,17], and metal element analysis [18,19]. Meng et al. determined the stable isotope of volatile compounds in wolfberry from Gansu, Ningxia and Qinghai by gas chromatography isotope ratio mass spectrometry (GC-IRMS), and combined this with one-way analysis of variance (ANOVA) for origin tracing, and reached a final accuracy 89.16%, 87.77%, and 85.87%, respectively [20]. Li et al. established the fingerprints of Zhejiang and Sichuan *Ophiopogon japonicus* using HPLC, an ultraviolet spectrophotometer (UV) and an evaporative light scattering detector (ELSD) [21]. After similarity analysis (SA), hierarchical cluster analysis (HCA), and principal component (PCA) analysis it was found that the fingerprint profiles of Zhejiang and Sichuan *Ophiopogon japonicus* had different similarities and characteristic peaks that could be effectively distinguished. Chromatographic and mass spectrometry combined with a chemometrics method has the advantages of high sensitivity, good stability, and reproducibility, but its operation process involves a large number of extraction and purification steps, which is complicated and difficult to operate [22]. DNA barcoding can trace origin based on the unique variant sequences of the same traditional Chinese medicine from different origins. This tracer technology does not require professional taxonomic knowledge and the detection is rapid, stable, and accurate, and it is one of the important methods for tracing the origin of Chinese herbs. DNA-barcoding technology has made great progress in the molecular identification of Chinese herbs, and has been included in the Pharmacopoeia of the People’s Republic of China. However, after concoction or other processing operations, the DNA of Chinese herbal medicines can be degraded, thus affecting identification [23].

Near-infrared (NIR) spectroscopy is a rapid on-line identification and analysis method that can scan solids directly and has the advantages of being fast, non-contact, and non-destructive [24]. However, NIR spectroscopy is rich in information that is both relevant and irrelevant, and which overlaps seriously [25]. Therefore, it is necessary to combine it with stoichiometric methods to analyze the source of traditional Chinese medicine [26]. Lv et al. traced 360 dendrobium from 12 different origins using NIR spectroscopy combined with stoichiometric analysis, with an accuracy of 91.85% [27]. Yu et al., introduced NIR combined with a support vector machine (SVM) to trace the origins of 81 Clinacanthus nutans samples from Hainan (China), Guangxi (China), and Malaysia, and the accuracy of the training set and test set were 96.36% and 95%, respectively [28]. Meng et al. tested a total of 90 oolong tea samples from three producing areas in Fujian province by NIR spectroscopy, and established a partial least square discriminant analysis (PLSDA) to identify the different spectral characteristics of different places, with a traceability accuracy of 89.3% [29]. NIR spectroscopy combined with interval combination one-verse-one partial least squares discriminant (IC-OVO-PLSDA) has been adopted to trace a total of 1120 Gastrodia elata samples from 14 different regions, and under the pretreatment of the standard normal variable (SNV), the total classification accuracy reached 92.5% [30].

Spectral data are susceptible to stray light, noise, baseline drift, and other factors, thus affecting modeling results [31]. The quality of pretreatment methods directly affects the accuracy of the model, so it is necessary to inquire into the influence of different pretreatment methods on origin tracing accuracy for *Ophiopogon japonicus*. There are many kinds of spectral pretreatment methods commonly used at present, such as first derivative (1D) [32], second derivative (2D) [33], Savitzky–Golay (S-G) smoothing [34,35], standard normal variable (SNV) transformation [36,37], multivariate scattering correction (MSC) [38,39], wavelet transform [40], orthogonal signal correction (OSC) [41,42], normalization [43], and standardization [44]. Different spectral pretreatment methods have their own advantages. A derivative algorithm can eliminate interference caused by baseline drift or smooth background, distinguish overlapping peaks, and improve resolution and sensitivity [45]. S-G smoothing can ameliorate the signal-to-noise ratio of spectrograms and reduce random noise [46]. SNV and MSC are used to eliminate the effect of scattering on the spectrum caused by different particle sizes and uneven particle distribution [47]. A wavelet transform can deduct the influence of instrument background or drift on the signal [48]. OSC can filter out irrelevant signals, simplify model data processing and improve model prediction ability [49]. Normalization and standardization can remove redundant data and enhance the differences between data. According to the effects of different preprocessing methods, we can divide them into four categories: baseline correction, scattering correction, smoothing, and scale scaling. Baseline correction includes first derivative and second derivative. Scattering correction includes MSC, SNV, OSC. Smoothing includes S-G smoothing, and scale scaling includes mean centering and area normalization [50].

Near-infrared spectroscopy combined with chemometrics analysis is an effective means of origin tracing, which has been confirmed by many studies. However, the existing research basically selects a single pretreatment method combined with a chemometric analysis method to build the origin tracing model. If the sample size is small, the establishment of a single model may reach a high or even 100% accuracy, but if the sample size is large, the accuracy of origin tracing may be slightly lower, with space for improvement. Therefore, choosing the best pretreatment method and suitable chemometrics method is particularly important. Based on the comparison of traditional single pretreatment methods, the best single pretreatment method was selected according to the results, combined with other effective methods to construct a combined pretreatment method, and used to assess the protected geographical indication of Zhejiang and non-Zhejiang *Ophiopogon japonicus* with chemometrics. PLSR, SVM and SIMCA were selected as pattern recognition methods to study the effects of different spectral pretreatment methods on classification performance and to build the best model for origin tracing.

## 2. Results and Discussion

### 2.1. NIR Spectra

**Figure 1:**
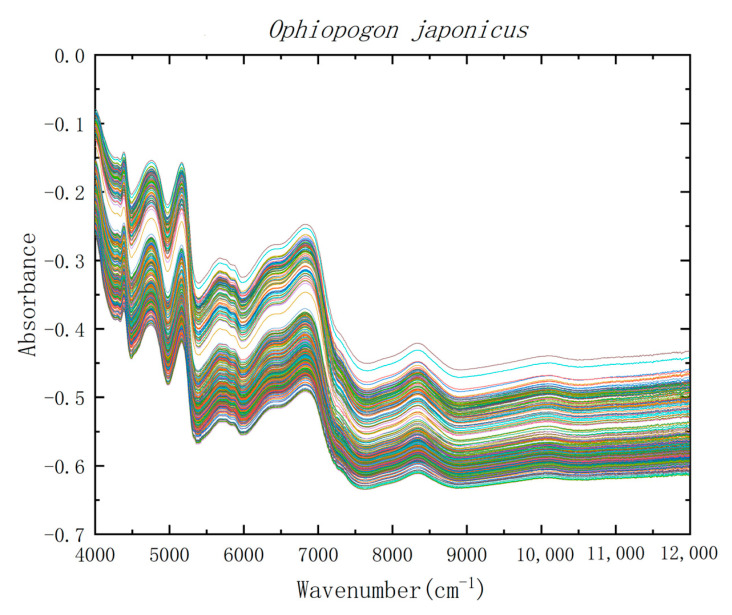
Raw spectra of Zhejiang and non-Zhejiang *Ophiopogon japonicus*.

NIR spectra were obtained by near-infrared spectrometer (Bruker Tensor 37, Germany). Near-infrared spectroscopy can be used for nondestructive testing, and the sample does not need to be cut or crushed. The samples of *Ophiopogon japonicus* were relatively small, and there would have been optical path loss if it had been placed on the near-infrared spectrometer optical fiber alone, so a cylindrical open quartz bottle with a diameter of 35 mm at the bottom and a height of 20 mm was customized. The quartz bottle was filled with *Ophiopogon japonicus*, and then placed on the optical fiber of the near-infrared spectrometer for detection. The original NIR spectra of *Ophiopogon japonicus* from different habitats showed very similar diffuse reflection patterns in the range of 4000–12,000 cm^−1^. Figure 1 shows the raw NIR spectra of Zhejiang and non-Zhejiang *Ophiopogon japonicus*.

It can be seen from Figure 1 that 4090–4396 and 4140–4450 cm^−1^ are the combination regions of stretching vibration and bending vibration of CH and CH_2_, respectively. 5620–5885 and 5680–6060 cm^−1^ are the first overtone regions of CH and CH_2_, respectively. Similarly, 6855–7020 and 7010–7288 cm^−1^ are their second overtone regions. The second overtone region of C=O stretching vibration appears at 5230–5370 cm^−1^. All absorption peaks may be caused by the steroid saponins, high isoflavones and polysaccharides in *Ophiopogon japonicus*. As can be seen from Figure 1, the NIR spectral shapes of Zhejiang and non-Zhejiang *Ophiopogon japonicus* were consistent, which could not be distinguished by the naked eye.

### 2.2. Preprocessing Method of NIR Spectra

**Figure 2:**
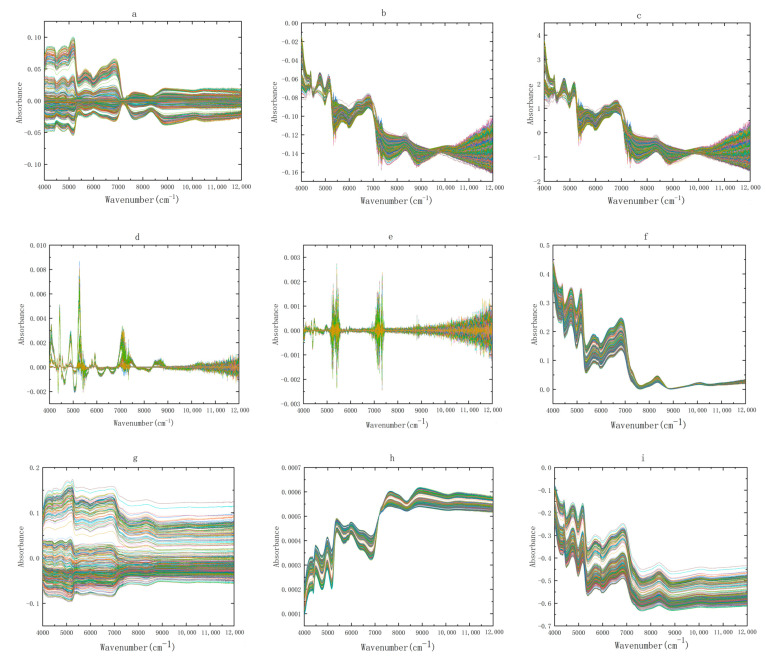
NIR spectra of Zhejiang *Ophiopogon japonicus* by nine preprocessing methods, (**a**) OSC, (**b**) MSC, (**c**) SNV, (**d**) first derivative, (**e**) second derivative, (**f**) baseline correction, (**g**) mean centering, (**h**) area normalization, and (**i**) Savitzky-Golay smoothing. Each color represents a sample of *Ophiopogon japonicus*.

**Figure 3:**
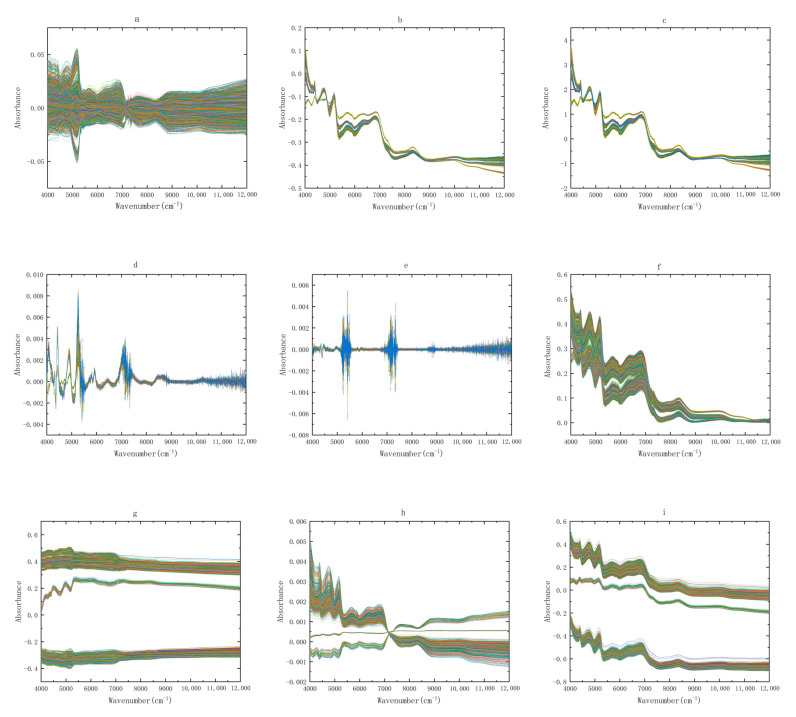
NIR spectra of non-Zhejiang *Ophiopogon japonicus* by nine preprocessing methods, (**a**) OSC, (**b**) MSC, (**c**) SNV, (**d**) first derivative, (**e**) second derivative, (**f**) baseline correction, (**g**) mean centering, (**h**) area normalization, and (**i**) Savitzky-Golay smoothing. Each color represents a sample of *Ophiopogon japonicus*.

Each NIR spectrogram has 2074 data points, and there are 3657 spectra, totaling 7,584,618 data points. The amount of data is very large and contains a lot of redundant information. Therefore, it is an effective method to select chemometrics to extract useful information from the spectra. The NIR spectra of Zhejiang and non-Zhejiang *Ophiopogon japonicus* after nine kinds of pretreatment are shown in Figure 2 and Figure 3, respectively. It can be seen from the below figure that the spectral coincidence degree after SNV and MSC pretreatment becomes higher, because they belong to scattering correction, which can eliminate the influence of scattering caused by uneven particle distribution or different particle size on the spectrum. The spectra after S-G smoothing show a significant reduction in noise, a decrease in error and an increase in signal-to-noise ratio. The spectra processed by the first and second derivative (1D and 2D) eliminates the interference caused by baseline drift and background smoothing, and can resolve overlapping peaks, improving resolution and sensitivity. After area normalization and mean centralization, the spectra may obviate the interference ascribed to size difference and different information structures. The spectra preprocessed by OSC filter out irrelevant signals, which can augment the prediction ability of the model.

### 2.3. Outlier Detection

**Figure 4:**
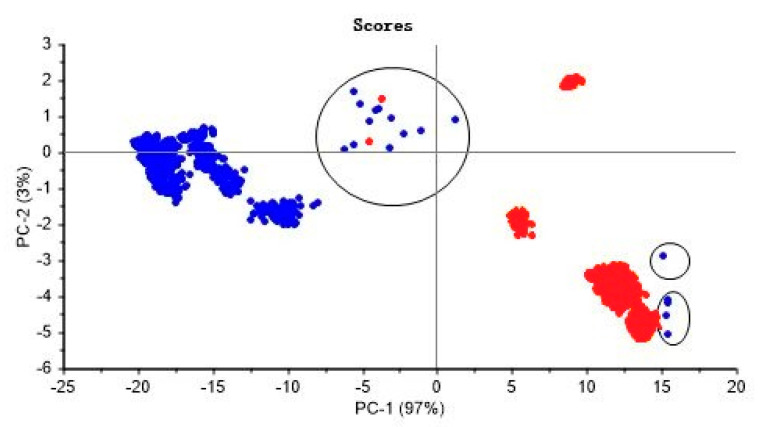
PCA scores and outliers of Zhejiang and non-Zhejiang *Ophiopogonis japonicus*. Blue represents Zhejiang samples, and red represents non-Zhejiang samples.

In this paper, PCA was chosen for outlier detection, and outliers were assessed by analyzing the high leverage (Hotelling T^2^ statistic) and residuals in the spectral data. In both cases, high-value samples with a significant level of 5% were considered as outliers [51,52]. The PCA results of Zhejiang and non-Zhejiang *Ophiopogon japonicus* are shown in Figure 4, with a total of 18 outlier samples, no. 26, 70, 189, 333, 545, 787, 789, 1453, 1672, 1834, 1990, 2366, 2370, 2678, 2881, 3021, 3451 and 3510, respectively.

### 2.4. Partial Least Squares Regression (PLSR)

**Figure 5:**
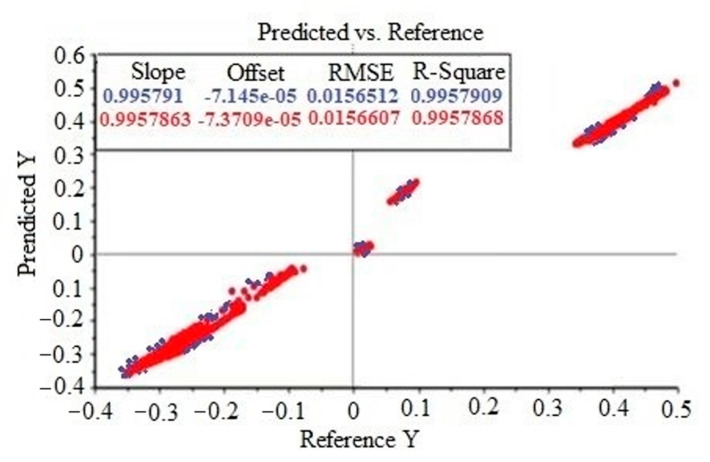
The R^2^ and RMSE of the raw NIR spectra of *Ophiopogon japonicus*, where blue is the actual value and red is the validation value.

The evaluation of the PLSR system depends on the following two important indicators: the coefficient of determination (R^2^) and the root mean square error (RMSE). The closer R^2^ is to 1, the better the prediction performance of the model is. R^2^ is greater than 0.91, indicating that the model meets the requirements. RMSE is used to calibrate, cross-validate, and predict during the analysis and evaluation of regression fit. The lower the RMSE value, the better the model prediction effect [53]. All the data of *Ophiopogon japonicus* were divided into the training and test sets according to a 3:7 ratio using Monte Carlo cross validation (MCCV). Figure 5 shows the results of the test set for the PLSR of *Ophiopogon japonicus*. Y is the dependent variable in PLSR analysis, representing the absorption obtained after near-infrared spectrum detection. The R^2^ and RMSE of the raw NIR spectra of *Ophiopogon japonicus* reached 0.995791 and 0.015651, respectively, indicating that the origin model of *Ophiopogon japonicus* was ideal and the prediction accuracy was high. It can be seen from Figure 5 that the actual values (blue data) and validation values (red data) of the R^2^ and RMSE are almost the same, which also explains the stability of the PLSR model.

Table 1 shows RMSE and R^2^ of PLSR evaluation indexes after single spectral pretreatment and combined spectral pretreatment. In the single spectral preprocessing method, the RMSE and R^2^ values of SNV, MSC, OSC and the first derivative are all greater than the original spectrum, indicating that these preprocessing methods can improve the prediction accuracy of the near-infrared tracing model. After the SNV pretreatment, R^2^ and RMSE both reached the optimal value. Therefore, we chose SNV as the basic method and combined FD, SD, S-G smoothing, and detrending to conduct the PLSR detection again. The results show that the accuracy of the combined pretreatment was higher than that of some single pretreatment methods, but SNV was still the best.

### 2.5. Support Vector Machine (SVM)

**Figure 6:**
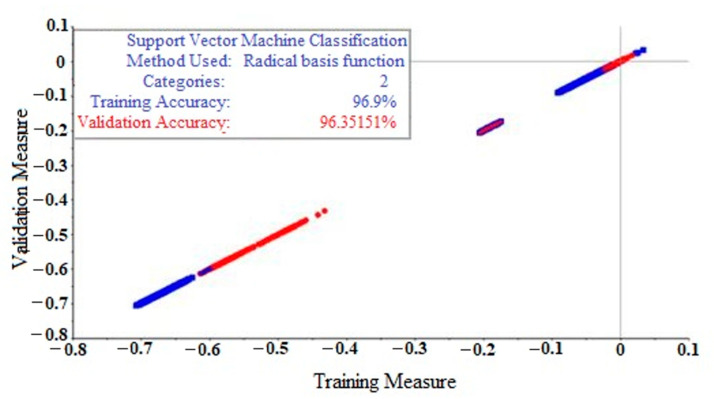
Accuracy of the training set of *Ophiopogon japonicus* with the SVM origin model, where blue is the actual value and red is the validation value.

**Figure 7:**
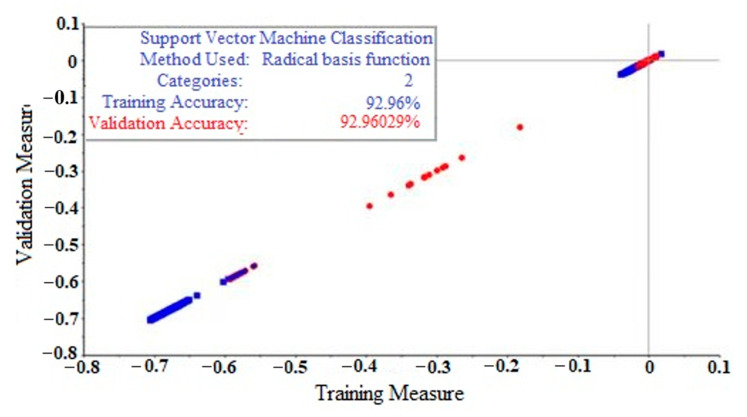
Accuracy of the testing set of *Ophiopogon japonicus* with SVM for the raw spectra, where blue is the actual value and red is the validation value.

The raw spectra of *Ophiopogon japonicus* were modeled and analyzed by SVM, and all data were divided into training and test sets by a 7:3 ratio according to MCCV. Figure 6 and Figure 7 show the SVM origin model diagram of raw spectra for the training and test sets, respectively, in which the accuracy of each set reached 96.90% and 92.96%, respectively. As can be seen from Figure 6, the actual and verified values of the training set accuracy based on the SVM model are basically the same, which are 96.90 and 96.35%, respectively, indicating that the training set accuracy of the SVM model is ideal. Similarly, the actual and verified values of the test set accuracy in Figure 7 are consistent with each other, both being 92.96%.

Table 2 shows the accuracy values of the training and testing sets after nine spectral pretreatments combined with SVM models. As can be seen from Table 2, after baseline correction, SNV, MSC and mean centralization, the accuracy of the training and testing sets has been significantly increased. After comparison, the accuracy of the training and testing sets could be increased to 99.73% and 98.40%, respectively, demonstrating that the SNV pretreatment method had the best effect. Although the second derivative algorithm can eliminate the interference caused by baseline and background to a certain extent, it also introduces some errors in the analysis process, which may reduce the accuracy of the training and test sets. On the basis of SNV, combined with first derivative, second derivative, S-G smoothing, and detrending, the results show that SNV + S-G smoothing has higher accuracy than other combination methods. However, SNV has the best effect. According to the calculation results of SNV, the accuracy, recall rate, and F1 scores of the SVM test set model reached 98.21%, 96.48% and 97.34%, respectively. This shows that the SVM model combined with the SNV pretreatment has good prediction performance.

### 2.6. Soft Independent Modeling of Class Analogies (SIMCA)

The soft independent modeling of class analogies (SIMCA) was used to model *Ophiopogon japonicus* samples, in which the training set accounted for 70% and the test set accounted for 30%. Table 3 shows the prediction accuracy of the training and test sets of the model, when nine different pretreatment methods are combined with SIMCA, respectively. As can be seen from Table 3, for the raw spectra, the accuracy of SIMCA for the training set of *Ophiopogon japonicus* could reach 85.76%, while the accuracy of the test set was only 54.53%. Both S-G smoothing reducing random errors and mean centralization deleting redundant data could enhance the difference between data, but did not improve the prediction accuracy for *Ophiopogon japonicus*. Area normalization, 1D and 2D, baseline correction, and OSC all failed to elevate the accuracy of the training and test sets. Among the nine pretreatment methods, only SNV and MSC could reach 100% accuracy for both the training set and the test set. From this, it can be seen that the factor affecting the accuracy of the training and test sets of *Ophiopogon japonicus* may be the spectral differences caused by the uneven particle sizes and non-uniform distribution of *Ophiopogon japonicus* grains.

**Figure 8:**
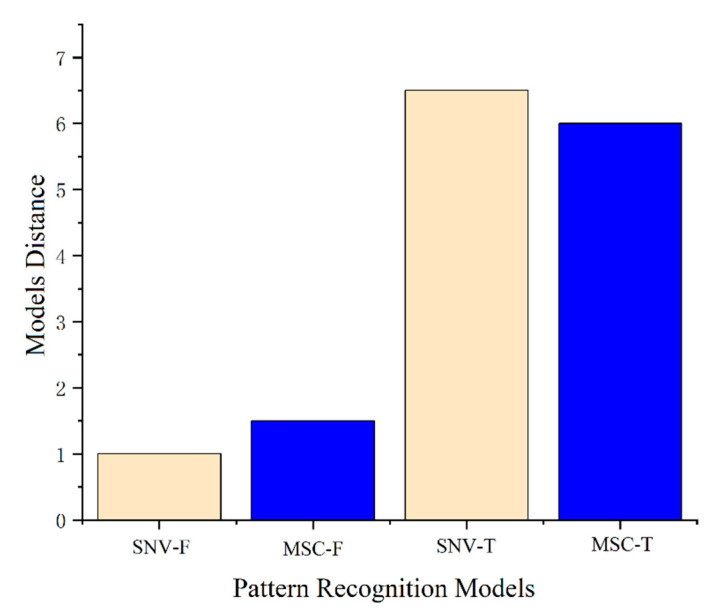
Distance graph between SIMCA classification models of Zhejiang and non-Zhejiang *Ophiopogon japonicus*, where T and F represent Zhejiang and non-Zhejiang, respectively. SNV-T, SNV-F, MSC-T and MSC-F designated the SIMCA model distances of Zhejiang and non-Zhejiang after SNV and MSC pretreatments, respectively.

In order to verify the performance of the model established by SNV and MSC combined with SIMCA, distance graphs between the models were used for validation. According to Shirzadifar et al., the formula of the SIMCA model distance is as follows:

$$D\left(r,g\right)=\sqrt{\frac{\sum_{k=1}^{p}(S_{k,r}^{2}\left(g\right)+S_{k,g}^{2}\left(r\right)}{\sum_{k=1}^{p}(S_{k,r}^{2}+S_{k,g}^{2})}}$$

where *r* and *g* represent SIMCA models of samples within the origin and outside the origin, respectively. *D*(*r*, *g*) represents the distance between *r* and *g*, and *k* represents the variable. *p* is the number of data matrices.$\;S_{k,r}^{2}\left(g\right)$ and $S_{k,g}^{2}\left(r\right)$ represent the standard deviations of distance between the samples in the two models. $S_{k}^{2}$ represents the residual variance of the test sample [54]. Figure 8 shows the distance between the SIMCA models of Zhejiang and non-Zhejiang *Ophiopogon japonicus*, where SNV-T and SNV-F represent the distance between SIMCA models of Zhejiang and non-Zhejiang Ophiopogon, respectively, after SNV pretreatment. Similarly, MSC-T and MSC-F were the same. The distance between SIMCA-SNV-T and SIMCA-SNV-F was 5.5, while the distance between SIMCA-MSC-T and SIMCA-MSC-F was 4.5. The distances between both the two classification models of SIMCA-SNV and SIMCA-MSC were all greater than 3, indicating that the models have good performance and can be correctly classified.

## 3. Materials and Methods

### 3.1. Ophiopogon japonicus Collection

A total of 2127 *Ophiopogonis japonicum* samples were collected from Zhejiang province, including 900 samples from Andong town of Cixi, 552 from Xinpu town of Cixi and 675 from Sanmen county of Taizhou. 1530 non-Zhejiang *Ophiopogonis japonicum* samples were collected from Sichuan, Hubei, Anhui and Shandong provinces, among which 765 were from Sichuan, 315 from Hubei, 225 from Anhui and 225 from Shandong. The *Ophiopogon japonicus* samples were put into a medicine washing machine, ultra-pure water was added so that *Ophiopogon japonicus* was submerged in water, and the machine was operated twice according to the standard operating procedures of the machine. The washed *Ophiopogon japonicus* samples were placed into an oven for dehydration, and were dried at 35 °C for 48 h. After washing and drying, all samples of *Ophiopogonis japonicum* were divided into the Zhejiang and non-Zhejiang categories.

### 3.2. Near-Infrared Spectroscopy Detection

The NIR spectra of *Ophiopogon japonicus* were collected by a Bruker Tensor 37 spectrometer in diffuse reflection mode. The samples were illuminated using a fiber optic bundle and scattered light was collected. The wave number of the original NIR spectrum ranged from 12,000 to 4000 cm^−1^. In the test conditions, the resolution and scanning times were 8 cm^−1^ and 64 cm^−1^, respectively.

### 3.3. Outlier Detection

Abnormal values refer to sample points that deviate significantly from other data in the sample, also known as outliers. For the class model, outliers in the training set could lead to model bias and affect the accuracy of modeling [55]. NIR spectra are high-dimensional and the presence of outliers can easily cause masking effects, so it is necessary to adopt appropriate methods to identify and eliminate the interference of outliers. The eigenvalue refers to the variance projected onto the axes after decomposition of the covariance matrix of the sample. The eigenvalues are distributed centrally on the axes, and outliers easily deviate from the axes, which is an important basis for PCA to detect outliers. The covariance matrix of the sample is projected onto the axis after decomposition. The eigenvalues are distributed intensively on the axis, and the abnormal eigenvalues easily deviate from the axis. Deviation from the axis is an important basis for PCA to detect outliers [56].

### 3.4. Data Preprocessing

In addition to the required basic sample characteristics, the spectral information collected by NIR spectrometer is often mixed with some irrelevant information, such as stray light and noise, which affects the accuracy of the modelling. Spectral preprocessing has the advantages of enhancing model representativeness and prediction ability, reducing random errors, ameliorating signal-to-noise ratio, resolving overlapping peaks, filtering out irrelevant information, and improving sensitivity. In this paper, nine pretreatment methods including mean centralization, area normalization, 1D, 2D, S-G smoothing, MSC, SNV, OSC and baseline correction were selected for spectral preprocessing of Zhejiang and non-Zhejiang *Ophiopogon japonicus*.

### 3.5. Three Different Chemometric Analysis

Partial least squares regression (PLSR) has been widely used in quantitative and qualitative analyses of NIR spectroscopy. The PLSR algorithm is a statistical method that uses latent variable modeling to observe the relationship between variable sets. PLSR is especially applicable in cases where there are more prediction matrices than observed variables and with the presence of multicollinearity in X values [57]. Let the original feature data X_0_ be N × m-dimensional data and Y_0_ be N × n-dimensional data. Let the first principal component axis vectors of X and Y be ω_1_ and C_1_, respectively, and the first pair of principal components t_1_ of X and u_1_ of Y be represented by ω1 and C1, where t_1_ = X × ω_1_, and u_1_ = Y × C_1_. The PLSR idea can be mathematically formalized as follows: maximize<Xω_1_, YC_1_>, subject to: $\|\omega_{1}\|=1$, $\|c_{1}\|=1$. In essence, PLSR is a low-rank approximation method to the original data space. Like the conjugate gradient method, one solution direction is determined each time according to certain criteria. On the basis of residual, orthogonal, or orthogonal conjugate decomposition is carried out constantly, and low-dimensional space is used to approximate the original space. Such dimensional reduction can overcome the latitude disaster and make the solution more stable and reliable [58].

The support vector machine (SVM) is an algorithm developed on the basis of statistical learning theory to solve machine learning problems. It has the advantage of high flexibility and can handle a large number of samples. SVM are widely used to solve regression problems and can create robust analysis models to effectively reduce the influence of outliers [59]. The basic idea of SVM comes from the optimal classification plane of linear discrimination, so as to improve the prediction ability and reduce the classification error rate [60].

Soft independent modeling of class analogies (SIMCA) is a pattern recognition method based on PCA. The classification of the whole sample is obtained after PCA analysis of the sample. On this basis, the corresponding class model of each sample is established, and then the unknown samples are reclassified according to the model, that is, the unknown samples are fitted with the class models of all samples respectively to determine the category [61]. The *k* vector of the class q is represented by the PCA model of the class, and the unknown sample P is fitted. Then the similarity between the unknown sample P and the class q model is represented by fitting residual. The population deviation and fitting residual of the class q model are used to calculate the critical value, and the classification of unknown samples is judged based on this [62].

## 4. Conclusions

NIR spectroscopy combined with chemometric analysis can be effective for tracing the origin of *Ophiopogon japonicus*. The effects of different spectral preprocessing methods on different pattern recognition methods were not the same after the removal of outliers. By comparing the RMSE and R^2^ of nine spectral preprocessing methods, it can be determined that SNV, MSC, OSC, first derivative, area normalization and baseline correction combined with PLSR can improve the prediction accuracy of the NIR tracing model, while second derivative, S-G smoothing and mean centering reduce the prediction accuracy. After selecting the combined pretreatment, the prediction accuracy of the PLSR model was significantly improved, but the model of SNV effect was still the best. After pretreatment with baseline correction, SNV, MSC and mean centering, the accuracy of the training and test sets of SVM was significantly improved, and reached the highest in SNV (99.73% and 98.40%, respectively), while the other five pretreatment methods failed to reach the ideal state. Therefore, on the basis of SNV, combined with other preprocessing methods, according to the results, the SNV + S-G smoothing model, the SNV + detrending model, and the SNV + first derivative + S-G smoothing model each have relatively high accuracy on the training set and test set, and the SNV model still has the best effect. Both the PLSR and SVM models show that the combined preprocessing method can improve the accuracy of the training set and test set, but it is not necessarily the best choice. It may be that too many pretreatment methods leads to an overfitting of the model. For the SIMCA model, among the nine pretreatment methods, only SNV and MSC could achieve 100% accuracy for both the training set and test set, which may be the best method for tracing the origin of *Ophiopogon japonicus*. The distance between SIMCA-SNV-T and SIMCA-SNV-F models was greater than three, indicating that the model has good performance and could be correctly classified, where T and F represent Zhejiang and non-Zhejiang *Ophiopogon japonicus*, respectively. The distance between the SIMCA-MSC models also illuminated that SIMCA could effectively distinguish Zhejiang and non-Zhejiang *Ophiopogon japonicus* after MSC pretreatment.

To sum up, this paper believes that NIR-SNV-SIMCA is a highly accurate origin tracing model, which is applicable to food, medicine and other fields. SNV is widely used in the pretreatment of solid and liquid samples, especially for non-uniform samples, and has universal applicability. According to the characteristics of the sample, different kinds of spectral pretreatment methods can also be selected, or different effects of pretreatment methods combined with chemometrics can be used to build an accurate and efficient origin tracing model.

## Figures and Tables

**Table 1:** RMSE and R^2^ of PLSR evaluation indexes after nine kinds of spectral pretreatment methods.

Pretreatment	RMSE	R^2^
Raw data	0.015651	0.995791
S-G smoothing	0.015648	0.995794
Area normalization	0.002146	0.994077
First derivative	0.003484	0.996229
Second derivative	0.003019	0.965649
Baseline correction	0.002928	0.989924
SNV	0.001438	0.997970
MSC	0.001812	0.997458
Mean centering	0.016034	0.977945
OSC	0.005945	0.997702
First derivative + SNV	0.002411	0.996878
Second derivative + SNV	0.003043	0.997842
S-G Smoothing + SNV	0.014972	0.995860
Detrending + SNV	0.005477	0.978463
SNV + detrending	0.001562	0.997657
SNV + First derivative	0.001529	0.997281
SNV + Second derivative	0.001617	0.986457
SNV + S-G smoothing	0.001498	0.997923
SNV + First derivative + S-G smoothing	0.001512	0.997346

**Table 2:** Accuracy of *Ophiopogon japonicus* sets based on SVM.

SVM	Training Set Accuracy	Testing Set Accuracy
Raw data	96.90%	92.96%
S-G smoothing	56.73%	57.40%
Area normalization	56.73%	57.40%
First derivative	56.73%	57.40%
Second derivative	89.86%	93.36%
Baseline correction	97.27%	96.90%
SNV	99.73%	98.40%
MSC	98.96%	97.90%
Mean centering	96.98%	95.96%
OSC	77.68%	91.16%
First derivative + SNV	78.56%	79.03%
Second derivative + SNV	92.75%	89.15%
S-G smoothing + SNV	65.97%	66.78%
Detrending+ SNV	80.38%	82.46%
SNV + Detrending	98.86%	98.73%
SNV + First derivative	96.43%	95.74%
SNV + Second derivative	90.17%	90.33%
SNV + S-G smoothing	99.65%	98.21%
SNV + First derivative + S-G smoothing	97.25%	98.57%

**Table 3:** Prediction accuracy of the training and testing sets of 9 different pretreatment methods combined with SIMCA respectively.

SIMCA	Training Set Accuracy	Testing Set Accuracy
Raw data	85.76%	54.53%
S-G smoothing	91.20%	52.81%
Area normalization	65.69%	67.40%
First derivative	58.95%	55.68%
Second derivative	71.54%	60.38%
Baseline correction	77.27%	76.90%
SNV	100.00%	100.00%
MSC	100.00%	100.00%
Mean centering	98.12%	43.51%
OSC	77.68%	81.16%

## Data Availability

Data is contained within the article.

## Sample Availability

Samples of the compounds are available from the authors.
